# Pre-cutting traction-assisted endoscopic submucosal dissection for rectal neuroendocrine tumors

**DOI:** 10.1055/a-2740-3701

**Published:** 2025-12-15

**Authors:** Qian Yang, Jia Xu, Jing Cao, Xiaowei Tang

**Affiliations:** 1 556508 Department of Gastroenterology, The Affiliated Hospital of Southwest Medical University, Luzhou, China

Endoscopic submucosal dissection (ESD) is standard for early gastrointestinal neoplasms 1 , yet bleeding risks persist. Pre-cutting traction techniques address this challenge 2 3 . We report successful rectal neuroendocrine tumor resection using ESD with pre-cutting traction, enabling safe complete dissection with enhanced submucosal visualization and reduced hemorrhage risk ( Video 1 ).

Demonstration of the establishment of the traction device, highlighting mucosal elevation at the lesion periphery via external thread traction. The technique optimizes submucosal visualization while enhancing procedural safety and reducing hemorrhage risk.Video 1

A 44-year-old woman who underwent ESD had a hemispherical, yellowish submucosal lesion at 10 cm from the anal verge ( Fig. 1 **a** ). Prior to mucosal incision, a traction technique was established: one end of a cotton thread was secured to the mucosa adjacent to the lesion using an endoclip, while a second endoclip anchored the thread to the contralateral mucosa, with the free end exiting the anal verge ( Fig. 1 **a** , Fig. 2 **a, b** ). External traction on the thread elevated the perilesional mucosa, optimizing submucosal exposure ( Fig. 1 **b** , Fig. 2 **c, d** ). Under this traction-enhanced view, a Dual knife was used to incise the mucosa and submucosa at marked points using ERBE Endocut Q (Effect 3) ( Fig. 2 **e** ). The knife was then advanced through the entry point for submucosal dissection ( Fig. 1 **c** ). During resection, sequential traction with endoclip and cotton thread provided dynamic field exposure, enabling stepwise whole lesion removal ( Fig. 1 **d** ). Hemostasis was achieved using a Coagrasper (Effect 4, 80W), followed by sucralfate spray for ulcer protection. The mucosal defect was closed with multiple hemoclips. Histopathological examination of the resected specimen confirmed a well-differentiated neuroendocrine tumor ( Fig. 3 ). The patient was discharged on postoperative day 3 without complications and remained asymptomatic during a 3-month follow-up endoscopy.

**Fig. 1:**
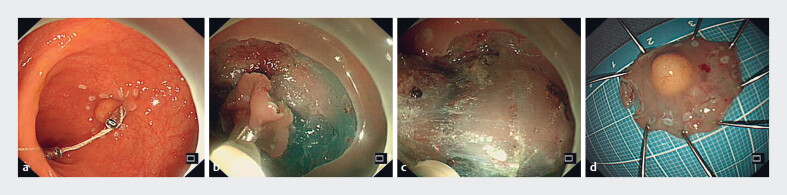
**a** Established traction system: thread-and-endoclip pre-cutting traction device ready for submucosal dissection. **b** Perilesional mucosal traction: targeted elevation optimizing submucosal plane visualization. **c** Submucosal dissection using Dual Knife. **d** Completely dissected lesion en bloc.

**Fig. 2:**
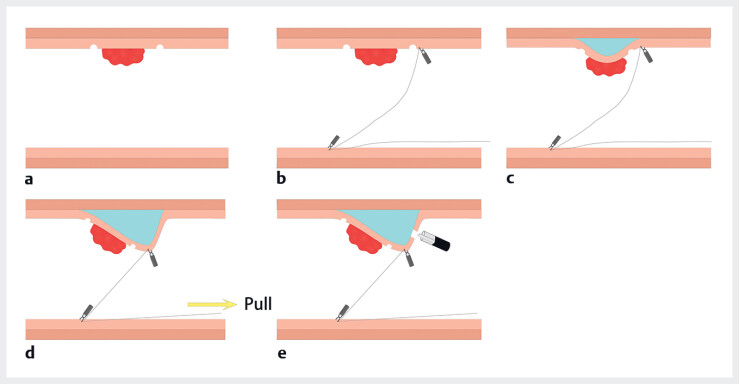
**a** The submucosal lesion. **b** Establishment of a thread-and-endoclip pre-cutting traction device adjacent to the lesion. **c** Administration of sodium hyaluronate-methylene blue-saline solution for mucosal lifting. **d** External traction on the free end of the thread to optimize submucosal exposure. **e** Mucosal incision with Dual Knife.

**Fig. 3:**
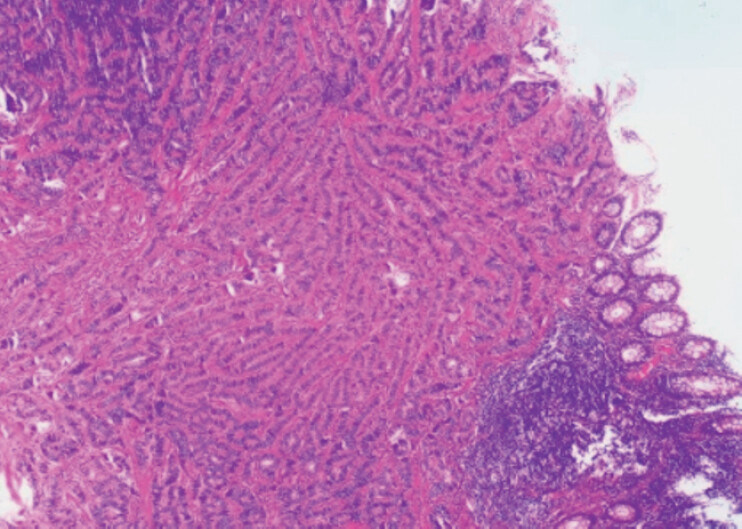
Histopathological examination confirmed a well-differentiated neuroendocrine tumor.

The key innovation established pre-cutting traction on perilesional mucosa before submucosal dissection. Crucially, this enabled: first, direct visual guidance; second, optimized submucosal exposure; third, widened mucosa–muscularis propria distance preventing mural injury. Collectively, this balances efficacy and safety in colorectal ESD.

Endoscopy_UCTN_Code_TTT_1AQ_2AD_3AD

## Notes

Endoscopy E-Videos https://eref.thieme.de/e-videos
*E-Videos* is an open access online section of the journal *Endoscopy* , reporting on interesting cases and new techniques in gastroenterological endoscopy. All papers include a high-quality video and are published with a Creative Commons CC-BY license. Endoscopy E-Videos qualify for HINARI discounts and waivers and eligibility is automatically checked during the submission process. We grant 100% waivers to articles whose corresponding authors are based in Group A countries and 50% waivers to those who are based in Group B countries as classified by Research4Life (see: https://www.research4life.org/access/eligibility/ ). This section has its own submission website at https://mc.manuscriptcentral.com/e-videos .
